# Mindfulness is associated with the weight status, severity of disease, and severity of extra-intestinal symptoms among individuals with irritable bowel syndrome

**DOI:** 10.3389/fpsyg.2025.1545033

**Published:** 2025-04-25

**Authors:** Vahideh Aghamohammadi, Hamed Rezakhani Moghadam, Esmail Najafi, Khadijeh Nasiri, Hanane Hamidi, Mohaddese Hajizadeh, Parastou Zamani, Neda Lotfi Yaghin, Hadi Bazyar, Farzad Najafipour

**Affiliations:** ^1^Department of Nutrition, Khalkhal University of Medical Science, Khalkhal, Iran; ^2^Student Research Committee, Khalkhal Faculty of Medical Sciences, Khalkhal, Iran; ^3^Department of Public Health, Khalkhal University of Medical Sciences, Khalkhal, Iran; ^4^Student Research Committee, Shiraz University of Medical Sciences, Shiraz, Iran; ^5^Department of Medical-Surgical Nursing, Khalkhal University of Medical Sciences, Khalkhal, Iran; ^6^Endocrine Research Center, Tabriz University of Medical Sciences, Tabriz, Iran; ^7^Department of Public Health, Sirjan School of Medical Sciences, Sirjan, Iran; ^8^Research Committee, Sirjan School of Medical Sciences, Sirjan, Iran

**Keywords:** irritable bowel syndrome, mindfulness, severity of diseases, obesity, Iran

## Abstract

**Objectives:**

Considering the high prevalence of irritable bowel syndrome (IBS), high medical costs, and the lack of complete treatment, paying attention to the psychological processes of these patients can lead to providing cognitive solutions to eliminate or reduce pain, and improve its consequences and psychological effects. Accordingly, the current study aims to evaluate the relationship between mindfulness and quality of life, IBS symptom severity, severity of extra-intestinal symptoms, and obesity among a cross-sectional sample of patients with IBS.

**Method:**

In this analytical cross-sectional study, 110 adults with IBS were confirmed according to Rome IV criteria by a physician. They were selected using cluster sampling. Various questionnaires and tools, including sociodemographic, physical activity, 24-item Five Facet Mindfulness Questionnaire Short Form (FFMQ–24), IBS-Quality of Life Instrument (IBS-QOL), IBS Symptom Severity Scale (IBS-SSS), and IBS Extra-intestinal Symptom Severity Scale (IBS-EISSS) were applied. SPSS software version 23 (IBM SPSS Statistics, Armonk, USA) was used for data analysis.

**Results:**

Inverse significant association was observed between the mindfulness score and BMI, Global IBS-SSS score, and Global IBS-EISSS score (*p* < 0.05). Moreover, the mindfulness score was inversely associated with weight in adjusted models (model 2: B = −0.16, *p* = 0.02; model 3: B = −0.21, *p* = 0.002). Mindfulness was associated with fewer odds of being overweight and obese in patients with IBS (OR = 0.93, CI: 0.87–0.98).

**Conclusion:**

Mindfulness had a significant relationship with obesity, the IBS symptom severity, as well as severity of extra-intestinal symptoms among those who suffer from IBS.

## Introduction

One of the functional disorders of the digestive system is irritable bowel syndrome (IBS) (Defrees and Bailey, 2017). IBS is a condition characterized by chronic abdominal pain in the absence of any structural organic disorder (Vicario et al., 2012). Individuals with IBS experience discomfort and chronic abdominal pain, along with changes in bowel habits, such as altered stool consistency, increased frequency of bowel movements, and symptoms such as bloating and the presence of mucus in stool (Mohagheghi et al., 2020). Beyond these physical symptoms, IBS is also strongly associated with psychological distress (Singh et al., 2018). Research indicates that individuals with IBS frequently experience high levels of anxiety, depression, and personality disorders, with a prevalence of psychological comorbidities reaching up to 54% (Tosic-Golubovic et al., 2010). This connection suggests that IBS is not merely a gastrointestinal disorder but also involves significant cognitive and emotional components. Given this interplay between physical and psychological factors, it is essential to explore how cognitive processes—such as mindfulness—may be altered in IBS patients (Jahangiri et al., 2017). Mindfulness means being present in the moment with whatever is happening now, without judgment and without expressing an opinion about what is happening; it means experiencing pure reality without explanation (Segal et al., 2019; Alamout et al., 2020). Mindfulness helps individuals understand that although negative emotions occur in life, they are not a fixed part of personality and life. This enables individuals to choose responses accompanied by contemplation and reflection instead of non-volitional reactions to these events (Keng et al., 2011). Based on this, mindfulness is a more efficient way to communicate with life, which leads to alleviating or relieving human pain and suffering, providing greater enrichment to life, and making it enjoyable (Siegel, 2010). According to scientific evidence, mindfulness has been effective in reducing anxiety and depression in patients with IBS (Mohamadi et al., 2015). Previous studies have found that higher levels of mindfulness in chronic pain patients were associated with lower self-reported pain and better pain-coping perceptions (Senders et al., 2018; Henriksson et al., 2016). The Five-Facet Mindfulness Questionnaire Short Form (FFMQ–24) is usually applied to evaluate mindfulness (Baer et al., 2006). However, a few studies have utilized this multidimensional approach to assess mindfulness in IBS patients. Moreover, considering the high prevalence of IBS, high medical costs, and the lack of complete treatment, paying attention to the psychological processes of these patients can lead to providing cognitive solutions to eliminate or reduce pain and improve its consequences and psychological effects. Accordingly, for the first time, the current study aims to evaluate the relationship between mindfulness and quality of life, IBS symptom severity, severity of extra-intestinal symptoms (including nausea/vomiting, early satiety, headache, back pain, fatigue, excessive throat gas, excessive intestinal gas, heartburn, urgency to defecate, straining to defecate, feeling of incomplete defecation, urgency to defecate urine, leg pain, muscle or joint pain, postprandial fullness), and obesity among a cross-sectional sample of patients with IBS. To the best of our knowledge, the association of mindfulness with the mentioned factors in patients with IBS has not been studied.

## Method

### Study design and participants

In this analytical cross-sectional study, 110 IBS patients confirmed by a physician were selected using simple cluster sampling. We randomly sampled each cluster (3 of 5 specialized gastroenterology clinics). IBS disease was diagnosed according to Rome IV criteria by a physician. Rome IV criteria include abdominal pain or discomfort lasting at least 4 days per month (or 1 day per week) in the past 3 months, accompanied by at least two to three of the following features: (1) Pain relieved with defecation (2) Association of onset of symptoms with changes in frequency of stool (3) Association of onset of symptoms with changes in stool form (Lacy and Patel, 2017). Based on the inclusion criteria, the patient group was recruited from specialized gastroenterology clinics in Ardabil City, Iran from September 2022 to March 2023.

The inclusion criteria for participation in the study were as follows:

- Confirmation of IBS,
- Adult patients (>18 years old)
- No pregnancy or lactation,
- Not suffering from any other gastrointestinal diseases such as celiac disease, Inflammatory bowel disease (IBD), abdominal surgery, and cancer, absence of warning signs (melena, rectal bleeding),
- Significant unintentional weight loss in the past 3 months,
- Not having diabetes,
- Not following a specific diet, psychological and social disorders or trauma,
- Willingness to participate.

The exclusion criteria were as follows:

- Lack of willingness to complete questionnaires.

The research protocol was in accordance with the guidelines of the Declaration of Helsinki. The Ethics Committee in Research of Khalkhal University of Medical Sciences approved the study protocol (Ethical code: IR.KHALUMS.REC.1400.017). The informed written consent form was completed for all subjects at the beginning of the study. Using the N = (Z _1-*α*/2_)^2^σ^2^/d^2^ formula and according to Eslampour et al. (2021)’s study, the sample size was calculated as 110 subjects.

### Measurements

A trained expert performed all measurements. Various questionnaires and tools, including sociodemographic, physical activity, mindfulness, IBS-Quality of Life Instrument (IBS-QOL), IBS Symptom severity scale (IBS-SSS), and IBS extra-intestinal symptom severity scale (IBS-EISSS) were applied. The demographic questionnaire involved the subjects’ sociodemographic information such as age, gender, education status, job, and marital status, consumption of alcoholic beverages, smoking, and medication. To evaluate physical activity (PA) the validated short form of the International Physical Activity Questionnaire (IPAQ) was used, and the results were reported as severe (>1,500 met-min/week), moderate (600–1,500 met-min/week), and low (<600 met-min/week) activity (Aadahl and Jørgensen, 2003). Dashti et al. (2014) have confirmed the validity of the Persian translation of the short form IPAQ (Cronbach’s alpha = 0.7).

In the current study, weight was measured using a digital scale made in Japan with an accuracy of 0.1 Kg, without shoes and with the least possible clothing. Height was estimated to the nearest 0.5 cm with a tape measure in a standing position, with shoulders in a normal alignment without shoes. BMI was calculated using the formula (weight in kilograms/height in square meters) (Heidari et al., 2020; Haidari et al., 2020).

### Mindfulness

The 24-item Five Facet Mindfulness Questionnaire Short Form (FFMQ–24) was used to evaluate mindfulness. This tool has 5 aspects: observing, describing, acting with awareness, non-judgment to inner experience, and non-reaction to inner experience. The scoring of each item is 1 to 5 (never or very rarely true - very often or always true). In this questionnaire, the questions that have the letter” R” are scored in reverse. The five aspects can be evaluated individually and combined as a total score. The total score range of FFMQ is from 39 to 195, and higher scores indicate higher levels of mindfulness. The validity of the FFMQ–24 Persian translation has been confirmed by Khanjani et al. (2022) (Cronbach’s alpha = 0.78). Internal consistency was acceptable for the current study (Cronbach’s *α* = 0.7).

### IBS-QOL questionnaire

The IBS-QOL questionnaire for patients with IBS has been translated into multiple languages and is used as an international tool. In the exploratory factor analysis of the final version of the questionnaire, 8 distinct factors were clearly distinguished: dysphoria, activities interference, body image, health worry, food abstinence, social reaction, sexual worry, and interpersonal relations. Both the 34-item IBS-QOL and the 36-item IBS-QOL are considered valid and widely used questionnaires. However, the construction of the 34-item IBS-QOL was part of an international effort and had a stronger methodology compared to the 36-item version. Furthermore, the 34-item version has been validated in more cultures and countries. The score range for this questionnaire is from 1 to 111. In a study by Jafari et al. (2013) Cronbach’s alpha Persian version of this scale was obtained for the examined sample at 0.88.

### IBS-SSS

It includes 4 questions that examine the symptoms of IBS, including pain, defecation disorder, bloating, the effect of the disease on daily life activities, and extraintestinal symptoms. The score of each section is a maximum of 100 and the total score of the questionnaire is a maximum of 500. Mild, moderate, and severe cases are classified based on scores of 75 to 175, 175 to 300, and above 300, respectively.

### IBS-EISSS

This questionnaire consists of 15 questions and aims to measure the severity of extra-intestinal symptoms including nausea/vomiting, early satiety, headache, back pain, fatigue, excessive throat gas, excessive intestinal gas, heartburn, urgency to defecate, straining to defecate, feeling of incomplete defecation, urgency to defecate urine, leg pain, muscle or joint pain, postprandial fullness. Each question has seven options (never, very low, low, sometimes, a lot, most of the time, and always), and the options “sometimes” to “always” are considered as the presence of a symptom. The final score of the questionnaire has changed from 0 to 100, where a higher score indicates more severe symptoms. The Persian version of the questionnaire has acceptable Cronbach’s alpha (0.84) and has been used in similar studies (Kheir-Abadi et al., 2010; Gholamrezaei et al., 2011).

### Statistical analysis

This study evaluated the normal distribution of the data using the Kolmogorov–Smirnov statistical test. The Kolmogorov–Smirnov test is used to test the null hypothesis that a set of data comes from a Normal distribution. Dependent variables were divided into quartiles based on mindfulness scores. The differences between the quartiles were evaluated using one-way analysis of variance (ANOVA) for quantitative variables (with LSD *post hoc*) and the chi-square test for categorical variables. ANOVA is used to determine whether there are any statistically significant differences between the means of three or more independent (unrelated) groups. The Chi-Square Test of Independence determines whether there is an association between categorical variables. Linear regression, along with modeling by adjusting for confounding factors (age, sex, physical activity, job, marital status, education, BMI, energy, smoking, alcohol, and medications) was used to determine the relationship between mindfulness (independent variable) and with anthropometric indices, global IBS score, global EISSS, and quality of life score (dependent variables). The Correlation between the mindfulness score (independent variable) and intensity of IBS (dependent variable) was assessed using the Pearson correlation test. Linear regression analysis is used to predict the value of a variable based on the value of another variable. Additionally, the Odds ratios (95% CI) for overweight and obesity according to the mindfulness score in the IBS patients compared to the reference (OR = 1) were measured using logistic regression, with adjustment of confounding factors (for age, sex, physical activity, job, marital status, education, BMI, energy, smoking, alcohol, and medications). Logistic regression is a data analysis technique that uses mathematics to find the relationships between two data factors. It then uses this relationship to predict the value of one of those factors based on the other. The prediction usually has a finite number of outcomes, like yes or no. In this study, a p-trend was calculated for each model to compare the ORs associated with quartiles in each model. SPSS software version 23 (IBM SPSS Statistics, Armonk, USA) was used for data analysis. A *p*-value of less than 0.05 was considered statistically significant.

## Results

The characteristics of subjects across quartiles of mindfulness scores are shown in Table 1. Significant differences were observed among the quartiles in terms of age (*p* = 0.002), education (*p* < 0.001), job (*p* < 0.001), physical activity (*p* = 0.003), medication (*p* = 0.001), smoking (*p* = 0.03), weight (*p* = 0.007), BMI (*p* < 0.001), Global IBS score (*p* = 0.008), Intensity of IBS (*p* = 0.002), and Global EIS score (*p* = 0.001). As observed, compared to individuals in quartile 4, those in quartile 1 had significantly more weight, BMI, and Global EIS scores (Table 1).

**Table 1:** The characteristics of subjects across quartiles of mindfulness score.

Characteristics mean (SD) or *N* (%)^1^	Mindfulness quartiles					*p*-value
	Q1 (*N* = 26)	Q2 (*N* = 30)	Q3 (*N* = 28)	Q4 (*N* = 26)	Total (*N* = 110)	
Mindfulness score	99.19 ± 6.08^a,b,c^	108.80 ± 1.68^d,e^	114.89 ± 2.75^f^	133.03 ± 7.68	113.0.80 ± 13.07	< 0.001^*^
Age (years)	39.73 ± 11.83^a^	30.30 ± 7.64^d^	40.57 ± 14.40^f^	34.15 ± 9.28	36.05 ± 11.71	0.002^*^
Sex (*N*) (%)						0.62^**^
Male	14 (53.8)	12 (40.0)	12 (42.9)	14 (53.8)	52 (47.3)	
Female	12 (46.2)	18 (60.0)	16 (57.1)	12 (46.2)	58 (52.7)	
Education (*N*) (%)						< 0.001^**^
Under-diploma	11 (42.3)	4 (13.3)	13 (46.4)	6 (23.1)	34 (30.9)	
Diploma	3 (11.5)	18 (60.0)	12 (42.9)	3 (11.5)	36 (32.7)	
Bachelor’s degree	12 (46.2)	8 (26.7)	3 (10.7)	14 (53.8)	37 (33.6)	
Master’s degree	0 (0.0)	0 (0.0)	0 (0.0)	3 (11.5)	3 (2.7)	
Job (*N*) (%)						< 0.001^**^
Unemployed	0 (0.0)	0 (0.0)	0 (0.0)	6 (23.1)	6 (5.5)	
Labor	0 (0.0)	7 (23.3)	3 (10.7)	3 (11.5)	13 (11.8)	
Housekeeper	10 (38.5)	15 (50.0)	15 (53.6)	10 (38.5)	50 (45.5)	
Government employee	8 (30.8)	4 (13.3)	3 (10.7)	4 (15.4)	19 (17.3)	
Non-government employee	4 (15.4)	0 (0.0)	0 (0.0)	3 (11.5)	7 (6.4)	
Freelance	4 (15.4)	4 (13.3)	7 (25.0)	0 (0.0)	15 (13.6)	
Marital status (*N*) (%)						0.10^**^
Single	4 (15.4)	14 (46.7)	9 (32.1)	9 (34.6)	36 (32.7)	
Married	22 (84.6)	16 (53.3)	19 (67.9)	17 (65.4)	74 (67.3)	
P.A (*N*) (%)						0.003^**^
Low	18 (69.2)	15 (50.0)	21 (75.0)	8 (30.8)	62 (56.4)	
Moderate	8 (30.8)	15 (50.0)	7 (25.0)	15 (57.7)	45 (40.9)	
High	0 (0.0)	0 (0.0)	0 (0.0)	3 (11.5)	3 (2.7)	
Weight (kg)	76.34 ± 10.73^a,c^	67.80 ± 13.35	73.17 ± 8.13	67.57 ± 10.14	71.13 ± 11.29	0.007 ^*^
BMI (kg/m^2^)	28.51 ± 3.71^a,b,c^	24.09 ± 3.32^d^	26.17 ± 2.39	25.47 ± 3.75	25.99 ± 3.65	< 0.001^*^
Type of BMI (*N*) (%)						0.12^**^
Normal	7 (26.9)	17 (56.7)	10 (35.7)	12 (46.2)	46 (41.8)	
High	19 (73.1)	13 (43.3)	18 (64.3)	14 (53.8)	64 (58.2)	
Medication (*N*) (%)						0.001^**^
Yes	19 (73.1)	8 (26.7)	10 (35.7)	7 (26.9)	44 (40.0)	
No	7 (26.9)	22 (73.3)	18 (64.3)	19 (73.1)	66 (60.0)	
Smoker (*N*) (%)						0.03^**^
Yes	4 (15.4)	0 (0.0)	4 (14.3)	0 (0.0)	8 (7.3)	
No	22 (84.6)	30 (100.0)	24 (85.7)	26 (100.0)	102 (92.7)	
Energy (kcal/d)	2805.02 ± 682.90	2812.80 ± 666.86	2845.40 ± 781.16	2812.55 ± 853.06	2819.20 ± 737.28	0.99^*^
Global IBS score	247.11 ± 96.52	279.16 ± 75.73^d,e^	200.89 ± 58.72	231.73 ± 109.21	240.45 ± 89.97	0.008^*^
Intensity of IBS (*N*) (%)						0.02^**^
Weak	4 (15.4)	4 (13.3)	6 (21.4)	9 (34.6)	23 (20.9)	
Moderate	15 (57.7)	15 (50.0)	21 (75.0)	9 (34.6)	60 (54.5)	
Severe	7 (26.9)	11 (36.7)	1 (3.6)	8 (30.8)	27 (24.5)	
Global EISSS score	45.38 ± 15.32^a,b,c^	38.86 ± 6.65^d,e^	26.14 ± 12.26	23.96 ± 13.32	33.64 ± 14.86	< 0.001^*^
Quality of life score	85.96 ± 16.99	82.76 ± 14.55	91.07 ± 14.33	89.65 ± 13.31	87.26 ± 15.00	0.14^*^

^1^Data are means ± SD for quantitative variables and frequency (percent) for qualitative variables.

*From ANOVA for quantitative variables, **Chi-square for qualitative variables.

Post hoc (LSD) according to the following pattern: ^a^Significant difference between 1 compared to 2. ^b^Significant difference between 1 compared to 3. ^c^Significant difference between 1 compared to 4. ^d^Significant difference between 2 compared to 3. ^e^Significant difference between 2 compared to 4. ^f^Significant difference between 3 compared to 4. Type of BMI: normal: BMI< 25 and high: BMI ≥ 25 (over weight and obese) Intensity of IBS: Low (75 – <175), moderate (≥175 – <300), severe (≥ 300–500).

BMI, body mass index; PA, physical activity; IBS, irritable bowel syndrome; EISSS, extra-intestinal symptom severity scale.

The correlation between the mindfulness score (independent variable) and the intensity of IBS (dependent variable) is presented in Table 2. Mindfulness scores correlated with the intensity of IBS before (Spearman test) and after the adjustment (the Partial test; with correction for age, sex, physical activity, job, marital status, education, BMI, energy, smoking, alcohol, and medications) (Model 1: *p* = 0.002; Model 2: *p* < 0.001) (Table 2).

**Table 2:** The correlation between the mindfulness score (independent variable) and IBS symptom severity (dependent variable).

Variable	Continuous mindfulness score	
	*R*	*p*-value
IBS symptom severity		
Model 1^a^	−0.28	0.002
Model 2^b^	−0.44	< 0.001

*p < 0.05 was considered as significant. R was considered as correlation coefficient.*

^a^Correlation and significant with the Spearman test (without adjustment).

^bCorrelation and significant with the Partial-test along with the adjustment of for age, sex, physical activity, job, marital status, education, BMI, energy, smoking, alcohol, and medications.^

The association between mindfulness score (independent variable) with anthropometric indices, global IBS score, global EIS score, and quality of life score (dependent variables) in the IBS patients is shown in Table 3. As presented in Table 3, in all models [(a) Model 1, linear regression analysis without adjustment; (b) Model 2, linear regression analysis with adjustment for age and sex; (c) Model 3, linear regression analysis with correction for age, sex, physical activity, job, marital status, education, BMI, energy, smoking, alcohol, and medications] inverse significant association was observed between the mindfulness score and BMI (model 1: *B* = −0.06, *p* = 0.02; model 2: *B* = −0.05, *p* = 0.01; model 3: *B* = −0.10, *p* < 0.001), Global IBS score (model 1: *B* = −2.28, model 2: *B* = −0.05; model 3: *B* = −0.10, *p* for all <0.001), and Global EIS score (model 1: *B* = −0.68; model 2: *B* = −0.67; model 3: *B* = −0.73, p for all <0.001). Moreover, the mindfulness score was inversely associated with weight in adjusted models (model 2: *B* = −0.16, *p* = 0.02; model 3: *B* = −0.21, *p* = 0.002). A positive but no significant association was observed between mindfulness and quality of life (*p* > 0.05) (Table 3).

**Table 3:** The association between mindfulness score (independent variable) with anthropometric indices, global IBS symptom score, global EISSS score, and quality of life score (dependent variables) in the IBS patients.

Variables	Model 1^a^				Model 2^b^				Model 3^c^			
	B (Unstandardized)	SE	Effect size	*p*-value	B (Unstandardized)	SE	Effect size	*p*-value	B (Unstandardized)	SE	Effect size	*p*-value
^a^Weight (kg)	−0.14	0.08	0.02	0.07	−0.16	0.07	0.31	0.02	−0.21	0.06	1	0.002
^a^BMI (kg/m^2^)	−0.06	0.02	0.04	0.02	−0.05	0.02	0.40	0.01	−0.10	0.02	1.12	< 0.001
Global IBS symptom score	−2.28	0.62	0.12	< 0.001	−2.33	0.63	0.12	< 0.001	−3.89	0.71	0.49	< 0.001
Global EISSS score	−0.68	0.08	0.56	< 0.001	−0.67	0.08	0.55	<0.001	−0.73	0.10	0.75	<0.001
Quality of life score	0.15	0.10	0.01	0.14	0.16	0.10	0.04	0.14	0.26	0.14	0.08	0.06

*p < 0.05 was considered as significant. aModel 1: linear regression analysis without adjustment. bModel 2: linear regression analysis with adjustment for age and sex. cModel 3: linear regression analysis with correction for age, sex, physical activity, job, marital status, education, BMI, energy, smoking, alcohol, and medications.*

^aBMI is not included in the model for these two variables.^

BMI, body mass index; IBS, irritable bowel syndrome; EISSS, extra-intestinal symptom severity scale.

F-square is effect size (> = 0.02 is small; > = 0.15 is medium; > = 0.35 is large).

Odds ratios (95% CI) for overweight and obesity according to the mindfulness score in the IBS patients are shown in Table 4. According to the continuous score of mindfulness, there was a significant negative association between mindfulness and overweight and obesity in adjusted model 3 (OR = 0.93, CI: 0.87–0.98). In other words, mindfulness was associated with fewer odds of being overweight and obese in patients with IBS (Table 4).

**Table 4:** Odds ratios (95% CI) for overweight and obesity according to the mindfulness score in the IBS patients.

Risk of overweight and obesity	Odds ratios (95% CI)	β	*p*-value
Model 1^a^	0.98 (0.96–1.01)	−0.01	0.41
Model 2^b^	0.99 (0.95–1.02)	−0.009	0.60
Model 3^c^	0.93 (0.87–0.98)	−0.07	0.01*

^*^p < 0.05 statistically significant by Multivariable logistic regression. ^a^Model 1: unadjusted. ^b^Model 2: adjusted for age and sex. ^c^Model 3: adjustment for age, sex, physical activity, job, marital status, education, BMI, energy, smoking, alcohol, and medications.

BMI, body mass index; IBS, irritable bowel syndrome.

Category of BMI: Overweight and obesity; BMI ≥ 25 and normal; BMI < 25.

## Discussion

Individuals with IBS often experience psychological distress, such as anxiety or depression, and they often report poorer quality of life (QoL) as the disease progresses (González-Moret et al., 2020). Research studies have shown that mindfulness meditation can decrease stress symptoms and can help patients with a range of chronic physical diseases (Bonhof et al., 2022; Greeson and Chin, 2019). The present study sought to better understand the relationship between mindfulness and the IBS symptom severity as well as the quality of life for those who suffer from the condition.

Our findings revealed a significant difference between mindfulness score quartiles with demographic characteristics and IBS symptoms. Also, we found an inverse significant association between mindfulness practice scores and IBS symptom scores. A positive correlation between mindfulness score and quality of life measures was also observed. Although, to the best of our knowledge, no study has explored the association of mindfulness with IBS symptom severity, these results are in agreement with those of other interventional studies in this regard. A study by Kearney et al. found that engaging in mindfulness-based stress reduction improved GI-specific anxiety and IBS-related quality of life (Kearney et al., 2011). In another randomized clinical trial, it was revealed that mindfulness-based cognitive therapy has the potential to improve quality of life and reduce IBS symptoms (Henrich et al., 2020).

In the IBS, it is supposed that impaired cognitive processes (memory, thinking, and attention) and dysfunctional thoughts act by developing neural connections between the brain and the gastrointestinal tract. This interaction disrupts emotional regulation and bowel functions which results in IBS symptoms (Enck et al., 2016). Compared to their healthy peers, people with IBS typically experience higher levels of anxiety, negative emotions, and poorer psychosocial well-being (Sirois, 2015; Jordan et al., 2016). de MendonÇa and colleagues reported that patients with IBS had depression, anxiety, mental tiredness, and lower quality of life (MendonÇa et al., 2020). Evidence shows a correlation between mindfulness and mental health indices, including high levels of positive affect, liveliness, adaptive emotional regulation, lower levels of negative affect, and psychological symptoms (Zernicke et al., 2012). Therefore, it can be deduced that a related mechanism by which mindfulness may help patients with IBS is by regulating the negative emotional effects. According to studies, mindfulness may act as a buffer to lessen the negative effects of emotion on mood and well-being (Wenzel et al., 2015). Furthermore, mindfulness and other emotion regulation techniques enhance parasympathetic nervous system activity and promote relaxation responses, which in turn diminish the levels of stress, the IBS symptom severity, and the rates of oxygen consumption and respiration (Mohammadi et al., 2021). It should also be noted that improved depressive symptoms might be associated with several healthy behaviors, including healthy diet, medication adherence, access to social support, physical activity, and other activities that lead to better disease outcomes (Forbes and Johnson, 2021).

Regarding the link between mindfulness score and anthropometric indices in patients with IBS, our results showed that a higher mindfulness score was associated with lower odds of overweight and obesity. In a study by Camilleri et al. (2015) women with higher dispositional mindfulness scores were less likely to be overweight and obese, and in men, higher mindfulness was associated only with lower odds of obesity. Also, Bektas and Gürkan (2023) reported that adolescents’ BMI had a significant negative correlation with their mindfulness, and mindfulness has a facilitating effect on the management of obesity. Mindfulness could be promising approach in addressing obesity by fostering healthier eating behaviors and improving psychological well-being and the relationship between mindfulness and weight can be explained by a number of mechanisms. Firstly, mindful eating encourages individuals to focus on the sensory experience of eating, which can lead to better food choices and reduced overeating (Kristeller and Wolever, 2011). It has been discovered that individuals with higher mindfulness scores report consuming fewer energy-dense food servings (Beshara et al., 2013). Another possible mechanism suggests that mindfulness decreases emotional eating or eating driven by external triggers decreasing emotional and binge eating (Jo and Yang, 2019; Morillo-Sarto et al., 2023; Lotfi Yagin et al., 2020). In line with this notion, a negative correlation between mindfulness scores with emotional and disordered eating has been documented (Kristeller et al., 2014). Moreover, increased awareness of hunger and satiety cues can lead to more controlled eating behaviors, as evidenced by significant reductions in energy intake and impulsive food choices (Kao et al., 2025). As mentioned previously, mindfulness can also help reduce chronic stress, which in turn, decreases abdominal adiposity. These results collectively imply that mindfulness reduces automatic and emotional reactions to food and the eating process (Morillo-Sarto et al., 2023).

It is also worth noting that mindfulness reduces IBS and obesity symptoms by modulating stress physiology (including inflammation and cortisol) while simultaneously improving cognitive-emotional processing and reducing psychological distress (Faria et al., 2023). Physiologically, practitioners observed declines in inflammatory markers (e.g., C-reactive protein) and a reduced cortisol awakening response, with one study noting altered brain activity in pain-related regions via functional MRI (Dalen et al., 2010). These results suggest that mindfulness may ease IBS and obesity symptoms through modulating stress physiology and refining cognitive and emotional processing (Daubenmier et al., 2011).

An additional issue that must be taken into account is that cultural dietary practices might shape individual responses to IBS interventions through distinct microbial compositions and dietary habits, though their impact on obesity outcomes across ethnic groups remains unclear (Haghbin et al., 2024). For example, one study of overweight patients with IBS-D noted a 46.21% reduction in IBS symptom severity scores, and another found that roughly 70% of overweight or obese women with IBS experienced symptom relief accompanied by weight loss through low FODMAP, prebiotic, and probiotic strategies (Linsalata et al., 2023). Khoo et al. (2023) documented that Malay, Chinese, and Indian patients display distinct microbial compositions and dietary habits, suggesting that cultural dietary patterns may influence adherence and biological responses (Khoo et al., 2023). In a separate study, adherence to a Mediterranean diet was linked with IBS symptom changes and gut microbiome alterations, although its effect on obesity outcomes was not specifically addressed (Chen et al., 2024). These findings indicate that while dietary interventions improve IBS symptoms broadly, cultural dietary practices appear to partly shape individual responses, with limited evidence to support clear ethnic differences in obesity reduction outcomes.

Although these findings should be interpreted with caution, this study has several strengths that distinguish it from existing literature. The study integrates mindfulness with various health outcomes, including weight status and extra-intestinal symptoms, which is less frequently explored in IBS research. By examining both psychological and physiological dimensions, it aligns with the growing recognition of the mind-gut connection in IBS management. Unlike many studies that primarily address gastrointestinal symptoms, this research highlights the impact of mindfulness on extra-intestinal manifestations, providing a broader understanding of IBS. The study employs a variety of validated scales to assess mindfulness, disease severity, and weight status, enhancing the reliability of findings. However, the limitations highlighted below necessitate cautious interpretation of results and underscore the need for more rigorous, diverse, and larger-scale studies to validate these findings. IBS is influenced by various psychological and physiological factors, complicating the isolation of mindfulness as a singular effective intervention. Also, the interplay between IBS symptoms and psychological disorders, such as anxiety and depression, complicates the assessment of mindfulness’s direct impact on disease severity and extra-intestinal symptoms. The absence of blinding also raises concerns about the validity of self-reported outcomes, potentially leading to the overestimation of mindfulness benefits. Finally, these findings are limited by the use of a cross-sectional design.

## Conclusion

Mindfulness has been shown to have a significant relationship with obesity and IBS symptom severity, as well as extra-intestinal symptoms. Integrating mindfulness into healthcare practices can enhance treatment strategies for obesity and IBS, promoting a healthier lifestyle and improved symptom management. Future research should explore personalized mindfulness interventions tailored to individual needs, focusing on the mind-gut connection and expanding accessibility to these therapies. Also, it is needed to refine mindfulness techniques and understand their long-term effects on diverse populations, ensuring that they are accessible and effective for all individuals seeking relief from these conditions.

## Data Availability

The raw data supporting the conclusions of this article will be made available by the authors, without undue reservation.
