# Supplementary material for: Mindfulness is associated with the weight status, severity of disease, and severity of extra-intestinal symptoms among individuals with irritable bowel syndrome
Source: Front Psychol. 2025 Apr 25;16:1545033. doi: 10.3389/fpsyg.2025.1545033 (PMC12061913; doi:10.3389/fpsyg.2025.1545033)
Supplement: Supplementary file 1 [file Data_Sheet_1.pdf]

Five Facet Mindfulness Questionnaire: Short-Form (FFMQ-SF)

Below is a collection of statements about your everyday experience. Using the 1–5 scale below, please indicate, in the box to the right of each statement, how frequently or infrequently you have had each experience in the past month (or other agreed time period). Please answer according to what really reflects your experience rather than what you think your experience should be.

1	2	3	4	5
Never or very rarely true	Not often true	Sometimes true, sometimes not true	Often true	Very often or always true

Item	Facet	Item score/ Reverse keyed items	Final score
1. I'm good at finding the words to describe my feelings.	DESCRIBE EXPERIENCES		
2. I can easily put my beliefs, opinions, and expectations into words.	DESCRIBE EXPERIENCES		
3. I watch my feelings without getting carried away by them.	NON-REACTIVITY		
4. I tell myself that I shouldn't be feeling the way I'm feeling.	SELF COMPASSION	6–	
5. It's hard for me to find the words to describe what I'm thinking.	DESCRIBE EXPERIENCES	6–	
6. I pay attention to physical experiences, such as the wind in my hair or sun on my face.	OBSERVING INNER EVENTS		
7. I make judgments about whether my thoughts are good or bad.	SELF-COMPASSION	6–	
8. I find it difficult to stay focused on what's happening in the present moment.	ACTING MINDFULLY	6–	
9. When I have distressing thoughts or images, I don't let myself be carried away by them.	NON-REACTIVITY		
10. Generally, I pay attention to sounds, such as clocks ticking, birds chirping, or cars passing.	OBSERVING INNER EVENTS		
11. When I feel something in my body, it's hard for me to find the right words to describe it.	DESCRIBE EXPERIENCES	6–	

12. It seems I am "running on automatic" without much awareness of what I'm doing.	ACTING MINDFULLY	6–	
13. When I have distressing thoughts or images, I feel calm soon after.	NON-REACTIVITY		
14. I tell myself I shouldn't be thinking the way I'm thinking.	SELF-COMPASSION	6–	
15. I notice the smells and aromas of things.	OBSERVING INNER EVENTS		
16. Even when I'm feeling terribly upset, I can find a way to put it into words.	DESCRIBE EXPERIENCES		
17. I rush through activities without being really attentive to them.	ACTING MINDFULLY	6–	
18. Usually, when I have distressing thoughts or images, I can just notice them without reacting.	NON-REACTIVITY		
19. I think some of my emotions are bad or inappropriate and I shouldn't feel them.	SELF-COMPASSION	6–	
20. I notice visual elements in art or nature, such as colors, shapes, textures, or patterns of light and shadow.	OBSERVING INNER EVENTS		
21. When I have distressing thoughts or images, I just notice them and let them go.	NON-REACTIVITY		
22. I do jobs or tasks automatically without being aware of what I'm doing.	ACTING MINDFULLY	6–	
23. I find myself doing things without paying attention.	ACTING MINDFULLY	6–	
24. I disapprove of myself when I have illogical ideas.	SELF-COMPASSION	6–	

This patient assessment measure is intended for clinical use in research and evaluation as a potentially useful tool to enhance an ACT-consistent approach. Not intended for commercial distribution. For use with Goubert DP, Törneke N, Purssey R, et al.: *Learning Acceptance and Commitment Therapy: The Essential Guide to the Process and Practice of Mindful Psychiatry*. Washington, DC, American Psychiatric Association Publishing, 2020.

FFMQ-SF Scoring Instructions

Record score and final score on survey. Record final facet scores in scoring table and sum. Divide sum by # items in facet. Record subscale mean. Note facet target(s).

	Facet scores/sum	Divide by	# Items in facet	Target(s)
Observing inner events			4	
Describe experience			5	
Non-reactivity			5	
Self-compassion			5	
Acting mindfully			5	
Total score				

This patient assessment measure is intended for clinical use in research and evaluation as a potentially useful tool to enhance an ACT-consistent approach. Not intended for commercial distribution. For use with Goubert DP, Törneke N, Purssey R, et al.: *Learning Acceptance and Commitment Therapy: The Essential Guide to the Process and Practice of Mindful Psychiatry*. Washington, DC, American Psychiatric Association Publishing, 2020.
