# Supplementary material for: Mindfulness is associated with the weight status, severity of disease, and severity of extra-intestinal symptoms among individuals with irritable bowel syndrome
Source: Front Psychol. 2025 Apr 25;16:1545033. doi: 10.3389/fpsyg.2025.1545033 (PMC12061913; doi:10.3389/fpsyg.2025.1545033)
Supplement: Supplementary file 2 [file Data_Sheet_2.pdf]

IBS extra-intestinal symptom severity scale (IBS-EISSS)								
	Item	never	very low	low	sometimes	a lot	most of the time	always
1	Nausea/vomiting							
2	Early satiety							
3	Headache							
4	Back pain							
5	Fatigue							
6	Excessive throat gas							
7	Excessive intestinal gas							
8	Heartburn							
9	Urgency to 11defecate							
10	Str12aining to defec13ate							
11	Feeling 14of incomplet15e defecation							
12	Urgency to defecate urine							
13	Leg pain							
14	Muscle or joint pain							
15	Postprandial fullness							
