# Supplementary material for: Mindfulness is associated with the weight status, severity of disease, and severity of extra-intestinal symptoms among individuals with irritable bowel syndrome
Source: Front Psychol. 2025 Apr 25;16:1545033. doi: 10.3389/fpsyg.2025.1545033 (PMC12061913; doi:10.3389/fpsyg.2025.1545033)
Supplement: Supplementary file 3 [file Data_Sheet_3.pdf]

08-30-10
QLW0156(IBSOS1)/06-15-10

IBS - SYMPTOM SEVERITY SCALE

IRRITABLE BOWEL SYNDROME STUDY

Patient Number	patid	Date of Study Participant Visit/Contact	visitm (mmm) / dd / yyyy
Protocol Number	study	Institution Code	instn
Form Week	week	*Seq No. seqno	**Step No. stepno
Key Operator Code	keyop		

This area completed by Clinic Staff only.

* Enter a '1' if this is the first of this form for this date. Designate subsequent forms on the same date with a 2, 3, etc.
** Enter the study participant's current study step number. Enter '1' if the study does not have multiple steps.

<u>INSTRUCTIONS</u>:
This form will help you describe the nature of your IBS. It is to be expected that your symptoms might vary over time, so please try and answer all the questions based on how you <u>currently</u> feel (i.e., over the last 10 days or so). All information will be kept in **strict** confidence.

1. For questions where a number of different responses are possible please circle the response appropriate to you.
2. Some questions will require you to write in an appropriate response.
3. Some questions require you to put a cross line which enables us to judge the severity of particular problem(s).

   For example:

   How severe was your pain?

   Please place your "x" anywhere on the line between 0 - 100% in order to indicate as accurately as possible the severity of your symptom.

   This example shows a severity of approximately 90%, with 100% representing "very severe" pain.

0% ——————————————————x— **100%**

**no pain**	**not very severe**	**quite severe**	**severe**	**very severe**

CONTINUE ON NEXT PAGE

IBS - SYMPTOM SEVERITY SCALE

Pt. No. ☐☐☐☐☐ *Seq. No. ☐☐ **Step No. ☐☐ Date ☐☐☐ mmm ☐☐ dd ☐☐☐☐ yyyy

For office use only: SCORE

1. Do you currently suffer from abdomen or belly pain? ……
   If No, go to 'b.'
   If Yes, continue.

   Check One
   Yes ☐ 1 No ☐ 2 mb901

   a. Indicate with an "**X**" on the line below the severity of your abdomen or belly pain:

   0% |——————————————| **100%**
   no pain — not very severe — quite severe — severe — very severe

   [mb902]

   b. Enter the number of days that you typically experience **abdominal pain *every 10 days:***
   (For example, if you enter 4, it means that you get pain 4 out of 10 days. If you get pain every day, enter 10.)

   Number of days with pain: ……………….. [mb903]

2. Do you currently suffer from abdominal distension? ………
   (bloating, swollen or tight tummy)
   (*Women, please ignore distension related to your periods.)
   If No, go question 3.
   If Yes, continue.

   Check One
   Yes ☐ 1 No ☐ 2 mb904

   a. Indicate with an "**X**" on the line below the severity of your abdominal distension/ tightness:

   0% |——————————————| **100%**
   no pain — not very severe — quite severe — severe — very severe

   [mb905]

3. Indicate with an "**X**" on the line below how satisfied you are with your bowel habits:

   0% |——————————————| **100%**
   not at all satisfied — not too satisfied — somewhat satisfied — very satisfied

   [mb906]

4. Indicate with an "**X**" on the line below how much your Irritable Bowel Syndrome affects or interferes with your life in general:

   0% |——————————————| **100%**
   not at all interferes — not much — quite a lot — completely interferes

   [mb907]

Date Form Keyed (DO NOT KEY): ________ / _______ / _______
