# Supplementary material for: Mindfulness is associated with the weight status, severity of disease, and severity of extra-intestinal symptoms among individuals with irritable bowel syndrome
Source: Front Psychol. 2025 Apr 25;16:1545033. doi: 10.3389/fpsyg.2025.1545033 (PMC12061913; doi:10.3389/fpsyg.2025.1545033)
Supplement: Supplementary file 4 [file Data_Sheet_4.pdf]

Information Sheet on the Irritable Bowel Syndrome-Quality of Life Measure (IBS-QOL)

Commonly Asked Questions

What is the IBS-QOL?

The IBS-QOL is a self-report quality-of-life measure specific to Irritable Bowel Syndrome (IBS) that can be used to assess the impact of IBS and its treatment. The IBS-QOL was developed using a needs based model.

Who developed the IBS-QOL?

The IBS-QOL was developed by a team of researchers from the University of Washington, Seattle under the direction of Dr. Donald L. Patrick and Dr. Douglas Drossman with sponsorship from Novartis Pharmaceuticals Corporation. Translations have been conducted by Health Research Associates under the direction of Mona Martin and MAPI Research Institute under the direction of Katrin Conway.

What are the IBS-QOL items?

The IBS-QOL consists of 34 items, each with a five-point response scale:

Items 1, 2, 4, 8-10, 12, 13, 16, 25-29, 34
1. Not at all   2. Slightly   3. Moderately   4. Quite a bit 5. Extremely

Items 3, 5-7, 11, 14, 15, 17-24, 30-33
1. Not at all   2. Slightly   3. Moderately   4. Quite a bit 5. A great deal

1. I feel helpless because of my bowel problems.
2. I am embarrassed by the smell caused by my bowel problems
3. I am bothered by how much time I spend on the toilet.
4. I feel vulnerable to other illnesses because of my bowel problems.
5. I feel fat because of my bowel problems.
6. I feel like I'm losing control of my life because of my bowel problems.
7. I feel my life is less enjoyable because of my bowel problems.
8. I feel uncomfortable when I talk about my bowel problems.
9. I feel depressed about my bowel problems.
10. I feel isolated from others because of my bowel problems.
11. I have to watch the amount of food I eat because of my bowel problems.
12. Because of my bowel problems, sexual activity is difficult for me.
13. I feel angry that I have bowel problems.
14. I feel like I irritate others because of my bowel problems
15. I worry that my bowel problems will get worse.
16. I feel irritable because of my bowel problems
17. I worry that people think I exaggerate my bowel problems.
18. I feel I get less done because of my bowel problems.
19. I have to avoid stressful situations because of my bowel problems
20. My bowel problems reduce my sexual desire.
21. My bowel problems limit what I can wear.

22. I have to avoid strenuous activity because of my bowel problems.
23. I have to watch the kind of food I eat because of my bowel problems.
24. Because of my bowel problems, I have difficulty being around people I do not know well.
25. I feel sluggish because of my bowel problems.
26. I feel unclean because of my bowel problems.
27. Long trips are difficult for me because of my bowel problems.
28. I feel frustrated that I cannot eat when I want because of my bowel problems.
29. It is important to be near a toilet because of my bowel problems.
30. My life revolves around my bowel problems.
31. I worry about losing control of my bowels
32. I fear that I won't be able to have a bowel movement.
33. My bowel problems are affecting my closest relationships
34. I feel that no one understands my bowel problems.

How is the IBS-QOL scored?

The individual responses to the 34 items are summed and averaged for a total score and then transformed to a 0-100 scale for ease of interpretation with higher scores indicating better IBS specific quality of life. There are also eight subscale scores for the IBS-QOL (Dysphoria, Interference with Activity, Body Image, Health Worry, Food Avoidance, Social Reaction, Sexual, Relationships).

The transformation formula used for the IBS-QOL total and scale scores is:

$$\text{Score} = \frac{\text{The sum of the items - lowest possible score}}{\text{Possible raw score range}} * 100$$

How is the IBS-QOL administered?

The IBS-QOL is designed to be self-administered, and takes an average of 10 minutes to complete. The IBS-QOL can be interviewer-administered if necessary.

What language versions of the IBS-QOL are available?

The IBS-QOL is available in the following languages:

Afrikaans	Arabic for Egypt	Chinese (Cantonese) for Hong-Kong)
Czech	Danish	Chinese (Mandarin) for China and Hong-Kong
Dutch (Belgium)	Dutch (Netherlands)	English (Australia)
English (Canada)	English (Ireland)	English (New Zealand)
English (South Africa)	English (UK)	English (USA)
Finnish	French (Belgium)	French (Canada)
French (France)	German (Austria)	German (Germany)
Hebrew	Hungarian	Italian
Korean	Norwegian	Spanish (Argentina)
Spanish (Chile)	Spanish (Columbia)	Spanish (Ecuador)
Spanish (Mexico)	Spanish (Peru)	Spanish (Spain)
Spanish (USA)	Swedish	

Is the IBS-QOL available for use?

For copies of the IBS-QOL, User's manual and scoring diskette, please contact:

Information Resources Centre
Mapi Research Institute
27, rue de la Villette
69 003 LYON
FRANCE
Tel: +33 (0) 4 72 13 66 67
Fax: +33 (0) 4 72 13 66 82
mpemery@mapi.fr

For further information or reprints, contact :

Donald L. Patrick
University of Washington
146 N. Canal Street, Suite 310
Seattle, Washington 98103
United States of America
Telephone number: (206) 685 7252
Facsimile number: (206) 616-3135
E-mail: donald@u.washington.edu
Website: www.seaqolgroup.org
